# Shear Wave Tensiometry Reveals an Age-Related Deficit in Triceps Surae Work at Slow and Fast Walking Speeds

**DOI:** 10.3389/fspor.2020.00069

**Published:** 2020-06-19

**Authors:** Anahid Ebrahimi, Jack A. Martin, Dylan G. Schmitz, Darryl G. Thelen

**Affiliations:** ^1^Department of Mechanical Engineering, University of Wisconsin-Madison, Madison, WI, United States; ^2^Department of Orthopedics and Rehabilitation, University of Wisconsin-Madison, Madison, WI, United States; ^3^Department of Biomedical Engineering, University of Wisconsin-Madison, Madison, WI, United States

**Keywords:** aging gait, Achilles tendon force, gastrocnemius, soleus, work loops, muscle-tendon unit

## Abstract

Prior studies have observed an age-related decline in net ankle power and work at faster walking speeds. However, the underlying changes in muscle-tendon behavior are not well-understood, and are challenging to infer from joint level analyses. This study used shear wave tensiometry to investigate the modulation of force and work done by the triceps surae across walking speeds. Fourteen healthy young (7F/7M, 26 ± 5 years) and older (7F/7M, 67 ± 5 years) adults were tested. Subjects walked on an instrumented treadmill at four walking speeds (0.75, 1.00, 1.25, and 1.50 m/s) while lower extremity kinematics and Achilles tendon shear wave speeds were collected. Subject-specific calibrations were used to compute Achilles tendon force from wave speed. Excursions of the soleus and gastrocnemius muscle-tendon units were computed from the kinematic data and subject-specific measures of the Achilles tendon moment arm. Work loop plots were then used to assess effective muscle-tendon stiffness during lengthening, and positive, negative, and net work production during stance. Two-way mixed ANOVAs were used to evaluate the effects of age group and walking speed on each outcome measure. Tendon loading during muscle-tendon lengthening (effective stiffness) did not differ between age groups, but did vary with speed. The soleus became effectively stiffer with increasing speed while the gastrocnemius became effectively more compliant. There was a marked age-related deficit in net soleus (−66% on average) and gastrocnemius (−36%) work across all walking speeds. We did not observe an age-speed interaction effect on net work production. These results suggest the age-related deficit in triceps surae output in walking is pervasive across speed, and hence seemingly not linked to absolute mechanical demands of the task.

## Introduction

Typical human walking relies heavily on power generation by the ankle plantar flexors (McGowan et al., 2008). For example, inverse dynamics analyses have found that the ankle accounts for 35–40% of the positive work generated at joints of the lower extremity during gait (Sawicki et al., 2009; Ebrahimi et al., 2017). Numerous studies have shown that ankle power (Winter et al., 1990; Cofré et al., 2011) and positive work (DeVita and Hortobagyi, 2000; Silder et al., 2008; Cofré et al., 2011; Crenna and Frigo, 2011) are diminished with aging, even when accounting for differences in step length. The decline in ankle power generation becomes more apparent at faster walking speeds (Judge et al., 1996; Kerrigan et al., 1998; Silder et al., 2008; Cofré et al., 2011).

Age-related changes in musculoskeletal properties and neural control likely contribute to the decline in distal power production. For example, older adults exhibit greater Achilles tendon compliance (Onambele et al., 2006; Stenroth et al., 2012), which can affect power generation by altering the operating lengths and contraction velocities of the triceps surae (Conway and Franz, 2020). In addition, older adults exhibit evidence of greater co-contraction about the ankle during stance (Schmitz et al., 2009), which could increase ankle stiffness while diminishing net ankle torque production. The use of co-contraction to stiffen the ankle joint could be a compensation for increased Achilles tendon compliance, lower muscle strength, and other age-related neuromotor impairments (Nagai et al., 2011). However, it remains challenging to infer individual muscle contributions from joint level analyses. For example, joint level analyses will underestimate the work done by muscle-tendon units when co-contraction is present. Shear wave tensiometry is a new non-invasive technology that facilitates measurements of muscle-tendon loading during locomotion (Martin et al., 2018; Keuler et al., 2019). Tensiometry measures of tendon loading can be coupled with measures of muscle-tendon kinematics to characterize the work done at the muscle level, and thereby provide additional insight into the underlying source of diminished ankle power in older adults.

In this study, we coupled shear wave tensiometry with subject-specific moment arm measures to characterize work-loops (Josephson, 1985; Biewener and Roberts, 2000; Dickinson et al., 2000; Biewener et al., 2004) of the triceps surae during walking in young and older adults. Work loops were used to evaluate the operating lengths, effective stiffness (Rouse et al., 2013), and work done by the gastrocnemius and soleus muscle-tendon units. We hypothesized that older adults would exhibit more negative gastrocnemius and soleus muscle-tendon work than young adults, which could arise from co-activation of the plantarflexors and dorsiflexors in mid-stance (Schmitz et al., 2009). Further, we expected to observe significantly lower positive work and net work generated by both muscle-tendon units in the older adults, with the age-related deficit amplified at faster walking speeds.

## Methods

### Subjects

Fourteen healthy young (7F, 26 ± 5 years, 1.77 ± 0.11 m, 73.96 ± 15.19 kg) and 14 healthy older (7F, 67 ± 5 years, 1.75 ± 0.06 m, 71.90 ± 12.21 kg) adults participated in this study. All subjects self-reported that they were able to comfortably walk on a treadmill, that they were without current (past 6 months) orthopedic or neurological impairment, and that they had no history of Achilles tendinopathy. The study protocol was approved by the University of Wisconsin-Madison Health Sciences Institutional Review Board. After providing written consent, all subjects initially walked for 5 min to acclimate to the treadmill and pre-condition the tendon (Hawkins et al., 2009). Subjects then walked on an instrumented treadmill at four speeds (0.75, 1.00, 1.25, and 1.50 m/s) while Achilles tendon shear wave speed, lower extremity kinematics (8 cameras, 190 Hz, Eagle cameras, Cortex software, Motion Analysis, Rohnert Park, CA) and ground reaction forces (Instrumented treadmill, 1,900 Hz, Bertec Corp., Columbus, OH) were collected.

Motion capture markers were placed on anatomical landmarks of the lower limb (1st and 5th metatarsal head locations over the shoe, medial and lateral malleoli, and medial and lateral femoral epicondyles) and as clusters on rigid plates secured to the thigh, shank and foot (Collins et al., 2009). Ankle and knee plantarflexion angles and torques were calculated via standard inverse kinematics and inverse dynamics calculations in Visual 3D using a 6-degree of freedom rigid body model (C-motion, Inc., Germantown, MD).

### Achilles Tendon Force Measurements

Shear wave tensiometry was used to measure tendon force, as described previously (Keuler et al., 2019). Briefly, a shear wave tensiometer, consisting of a custom tapping device and accelerometer array in series, was secured over the right Achilles tendon of all participants with self-adherent wrap. The piezo-actuated (PK4JQP2, Thorlabs, Newton, NJ) tapping device was driven throughout the duration of each collection by a 50 Hz square wave via an open-loop piezo controller (MDT694B, Thorlabs, Newton, NJ). The accelerometer array consisted of two miniature accelerometers (Model 352C23, PCB Piezotronics, Depew, NY) mounted 10 mm apart in a silicone mold (Mold Star 15 SLOW, Smooth-On, Macungie, PA). Accelerometery data were collected at 100 kHz and then bandpass filtered using a second-order, zero-lag Butterworth filter with 150 and 5,000 Hz cutoff frequencies. For each tap (rising edge of the square wave), we computed the time between wave arrival at the two accelerometers by finding the delay that maximized the normalized cross-correlation of the first and second accelerometer signals over a 1 ms window after the tap event. Sub-sample interpolation was performed using a local 3-point cosine fit of the normalized cross-correlation values (Cespedes, 1995). Shear wave speed was calculated by dividing the distance (10 mm) between the accelerometers by the time delay. Performing this analysis for each tap resulted in a 50 Hz tendon wave speed signal.

Subject-specific shear wave speed-force calibration was performed independent of the walking tasks. To do this, we first measured each subject's Achilles tendon moment arm, *r(*θ*)*, as a function of ankle angle θ. Moment arms were measured during passive ankle dorsiflexion by simultaneously tracking the ankle functional rotation axis via motion analysis and the Achilles tendon line of action using cine ultrasound (19 Hz, SonixTOUCH Research, BK Medical, Peabody, MA) (Keuler et al., 2019). Subjects were then asked to stand on an in-ground force plate (1,900 Hz, BP400600-2000, AMTI, Watertown, MA) and cyclically sway in the anteroposterior direction at 0.5 Hz, with rate guided by a metronome. Achilles tendon shear wave speed, lower extremity kinematics and force plate data were simultaneously collected during the sway tasks. Inverse dynamics analysis was used to compute the ankle torque *T* from the kinematics and forceplate data. Achilles tendon force, *F*_*cal*_, was then computed assuming that ankle torque was generated purely via the Achilles tendon, i.e., *F*_*cal*_ = *T*/*r*(θ). Tibialis anterior (TA) electromyographic signals (Trigno™, DelSys, Inc. Boston, MA) were recorded during the sway tasks and used to identify and remove periods of co-contraction, which typically occurred when subjects leaned backwards. Calibration slopes were obtained by performing a linear fit between tendon wave speed squared and *F*_cal_. Additional details of our calibration procedures and results were previously reported (Ebrahimi et al., 2020).

### Work-Loop Analysis

Tendon forces and excursions were calculated from the tendon wave speed and joint angle data, respectively. Analysis was performed on a minimum of 4 strides for each of the four walking speeds. Achilles tendon force (*F*) was computed using a prediction model of the form: $F=\beta \left(c^{2}-c_{\min}^{2}\right)$, where β is the slope of the subject-specific calibration, *c* is wave speed, and *c*_min_ is the minimum wave speed measured over all walking trials for a given individual. *c*_min_ is assumed to represent a zero-load state. The proportion of Achilles tendon force, normalized to body mass, partitioned to the gastrocnemius (35%, both medial and lateral heads) and soleus (65%) was based on relative physiological cross-sectional areas as measured via magnetic resonance imaging and assumed fiber lengths (Handsfield et al., 2014).

**Figure 1:**
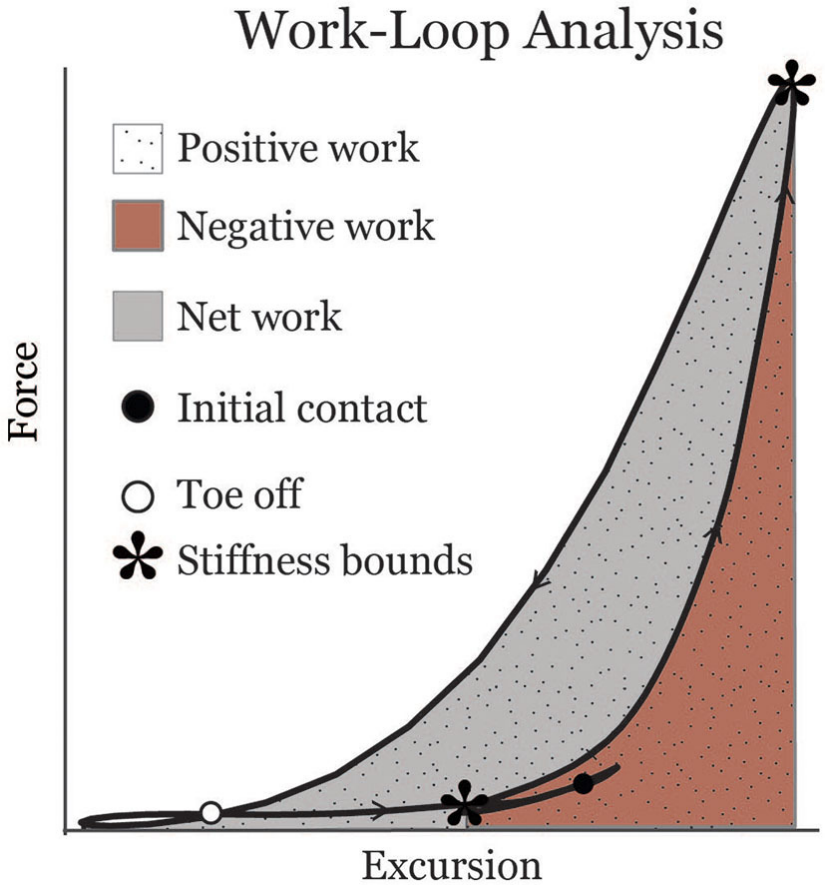
Representative soleus muscle-tendon work-loop displaying metrics analyzed in this study. Asterisks indicate bounds from which an effective stiffness measure was calculated.

Muscle-tendon excursion was defined as the muscle-tendon length relative to its length in an upright posture. Soleus excursion represented the change in length due to ankle rotation, and was computed by integrating the subject-specific Achilles tendon moment arm with respect to the ankle dorsiflexion angle during gait. The gastrocnemius excursion represented the change in length due to both ankle and knee rotation. The change in gastrocnemius length due to knee flexion was computed by integrating an average medial gastrocnemius moment arm-angle curve (Buford et al., 1997) with respect to the knee flexion angle during gait. Soleus and gastrocnemius work loops were created by plotting force vs. muscle-tendon excursion for each muscle (Figure 1). Muscle-tendon power was calculated as the time derivative of excursion multiplied by force. Work was calculated over the interval from the minimum excursion during early stance (corresponding to the inflection point after loading response when the muscle-tendon begins to lengthen) to the minimum muscle-tendon unit length in initial swing. Positive and negative work values were calculated by integrating the positive and negative portions of the power curve, respectively, over this interval. Positive and negative work were summed to estimate net work (shaded area in Figure 1). Effective stiffness of the muscle-tendon unit was calculated as the slope of the force-excursion plot from the instance of minimum excursion during early stance to maximum excursion (asterisks in Figure 1). Total excursion was defined as the difference between maximum excursion and minimum excursion.

Two-way mixed ANOVAs with Bonferroni *post-hoc* corrections were used to evaluate the effects of age group and walking speed on work, effective stiffness, and excursion for the gastrocnemius and soleus (*p* = 0.05 significance). Mean values are presented with standard deviations in parentheses [i.e., mean (SD)].

## Results

### Work

**Figure 2:**
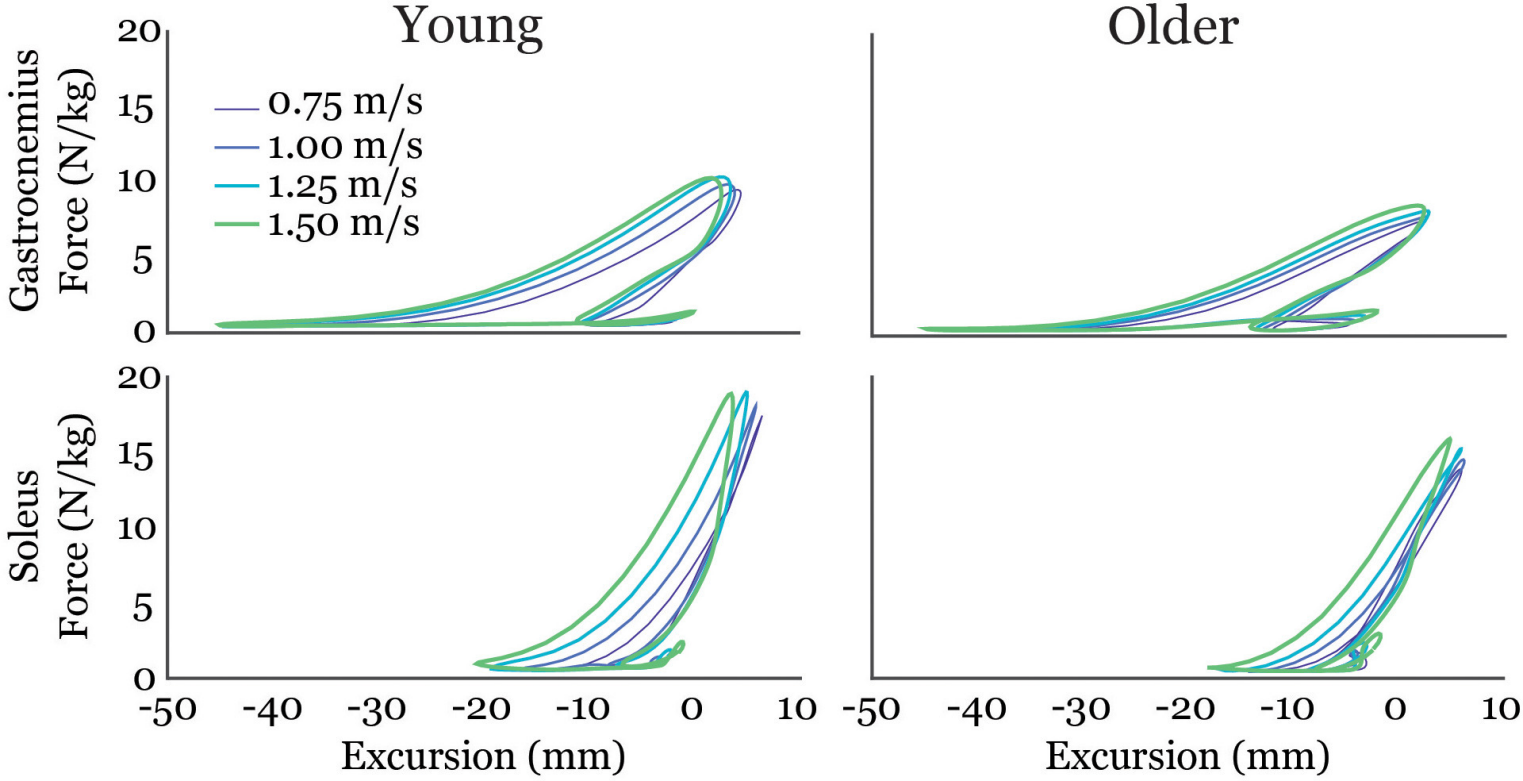
Average gastrocnemius (top row) and soleus (bottom row) work-loops for young and older adults at each of the four walking speeds tested. Excursion of each muscle-tendon unit was defined as the muscle length relative to its length in an upright posture.

**Figure 3:**
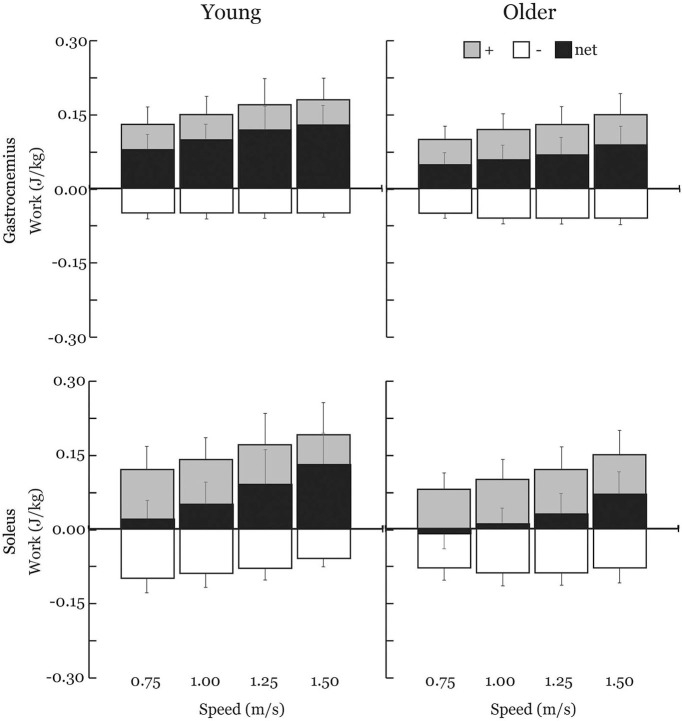
Average positive (+), negative (–), and net muscle-tendon work across walking speeds with one-directional standard deviation error bars. Older adults exhibited substantial reductions in net work production by both the soleus (−66%) and the gastrocnemius (−36%) across all walking speeds. Positive and net work significantly increased with speed (main speed effect) and were significantly lower in older adults (main age effect) for both the gastrocnemius and soleus. Negative work exhibited an age-by-speed interaction for the soleus; only young adults did less negative work with increasing speed. Negative work was not different between young and older adults for the gastrocnemius.

Gastrocnemius (gas) and soleus (sol) work loops varied significantly with speed and between age groups (Figure 2). Muscle-tendon loading patterns during stretch were relatively invariant with walking speed for both groups. However, the subsequent loading during shortening was substantially extended with increasing walking speeds, particularly in the soleus. On average, older adults exhibited muscle-tendon work loops that were characterized by lower force magnitudes, and less positive work in shortening. Statistically, net work was significantly increased with speed (main speed effect, gas, sol: *p* < 0.001) and was lower in older adults (main age effect, gas, sol: *p* < 0.005) (Figure 3, Table 1). The age-related deficit in net work was in part due to a significant (main age effect, gas: *p* = 0.03, sol = 0.02) deficit in positive work. Positive work increased with speed (main speed effect, gas, sol: *p* < 0.001). No age-speed interactions were observed in net or positive work for either muscle. However, negative work exhibited an age-by-speed interaction (sol: *p* < 0.002) for the soleus. Young adults did progressively less negative work with increasing speed, as determined by *post-hoc* comparisons. However, in the older adults, negative soleus work was relatively constant across speeds, with a small reduction present only at the fastest walking speed (1.50 m/s) compared to the other speeds. For the gastrocnemius, negative work exhibited a speed effect but not an age effect (main speed effect, gas: *p* < 0.001; main age effect, gas: *p* = 0.14).

**Table 1:** Net muscle-tendon work (J/kg) significantly increased with walking speed (main speed effect, gastrocnemius, soleus: *p* < 0.001) and was lower in older adults (main age effect, gastrocnemius, soleus: *p* < 0.005).

**Speed (m/s)**	**GASTROCNEMIUS**		**Soleus**	
	**Young**	**Older**	**Young**	**Older**
0.75	0.08 (0.03)	0.05 (0.02)	0.02 (0.04)	−0.01 (0.03)
1.00	0.10 (0.03)	0.06 (0.03)	0.05 (0.04)	0.01 (0.03)
1.25	0.12 (0.05)	0.07 (0.03)	0.09 (0.07)	0.03 (0.04)
1.50	0.13 (0.04)	0.09 (0.04)	0.13 (0.06)	0.07 (0.04)

*We did not observe a significant interaction effect (gastrocnemius: p = 0.35, soleus: p = 0.05)*.

### Effective Stiffness and Total Excursion

The relationship between tendon loading and muscle-tendon excursion showed a strong linear relationship (sol: *R*^2^ = 0.87 ± 0.08, range: 0.58–0.98; gas: *R*^2^ = 0.90 ± 0.06, range: 0.74–0.98). There were no significant differences in effective stiffness with age (group main effect, gas: *p* = 0.18, sol: *p* = 0.24) (Table 2). However, there was a significant increase in soleus effective stiffness with walking speed (speed main effect, sol: *p* = 0.02) and a significant decrease in gastrocnemius stiffness with walking speed (speed main effect, gas: *p* < 0.001). The total excursion increased with speed (main speed effect, gas, sol: *p* < 0.001) but did not differ between age groups (main age effect, gas, sol: *p* = 0.29) (Table 3).

**Table 2:** Effective muscle-tendon stiffness (N•kg^−1^•mm^−1^) did not significantly differ with age (group main effect, gastrocnemius: *p* = 0.18, soleus: *p* = 0.24).

**Speed (m/s)**	**GASTROCNEMIUS**		**Soleus**	
	**Young**	**Older**	**Young**	**Older**
0.75	0.59 (0.18)	0.50 (0.24)	1.18 (0.37)	1.07 (0.50)
1.00	0.55 (0.14)	0.46 (0.20)	1.23 (0.35)	1.04 (0.40)
1.25	0.53 (0.12)	0.43 (0.20)	1.31 (0.35)	1.07 (0.43)
1.50	0.50 (0.11)	0.41 (0.20)	1.33 (0.34)	1.15 (0.51)

*The soleus effective stiffness increased (speed main effect, p = 0.02) while the gastrocnemius stiffness decreased with walking speed (speed main effect, p < 0.001). We did not observe a significant interaction effect (gastrocnemius: p = 0.84, soleus: p = 0.39)*.

**Table 3:** Total muscle-tendon excursion (mm) significantly increased with speed (main speed effect, gas, sol: *p* < 0.001) but did not differ between age groups (main age effect, gas, sol: *p* = 0.29).

**Speed (m/s)**	**GASTROCNEMIUS**		**Soleus**	
	**Young**	**Older**	**Young**	**Older**
0.75	43.08 (4.09)	41.29 (5.78)	21.26 (3.81)	19.64 (5.36)
1.00	47.02 (3.56)	44.79 (5.34)	23.51 (3.49)	21.72 (5.33)
1.25	49.47 (3.55)	47.41 (5.41)	25.74 (4.23)	23.61 (5.66)
1.50	49.32 (3.62)	48.46 (4.97)	26.05 (4.24)	24.30 (5.18)

*We did not observe a significant interaction effect (gastrocnemius: p = 0.54, soleus: p = 0.90)*.

## Discussion

This study leveraged shear wave tensiometry to characterize the work done by the triceps surae during gait. We show that muscle-tendon lengthening behavior (effective stiffness) varies with speed, but is generally similar between age groups. However, we observed a marked age-related deficit in net soleus (−66% on average) and gastrocnemius (−36%) work across all walking speeds. The soleus results are consistent with prior reports of an age-related deficit in ankle power, though joint level analyses have often only found significant deficits at faster walking speeds (Silder et al., 2008). Notably, we did not observe an age-speed interaction effect on work; suggesting the age-related deficit in triceps surae output in walking is pervasive and not linked to increasing mechanical demands of the task.

The effective stiffness exhibited by the triceps surae was dependent on speed, but did not vary with age. Gastrocnemius stiffness decreased with walking speed, reflecting greater compliance at the muscle-tendon level. The increased compliance is attributable to gastrocnemius shortening with knee flexion in stance, which increases with walking speed. Both young and older adults exhibit greater effective soleus stiffness with increasing speed, a result that is consistent with ankle stiffness. Effective ankle stiffness, based on the slope of the moment-angle curve, also increases with speed (Frigo et al., 1996; Shamaei et al., 2013) but exhibits no difference between young and older adults walking at the same normalized speed (Crenna and Frigo, 2011). Another study found effective ankle stiffness did not correlate with age in a group of older adult women (65–91 years old) (Collins et al., 2018). Interestingly, the structural behavior of older adult tendon has been shown to differ from young adults, with there being an age-related increase in tendon compliance (Onambele et al., 2006; Stenroth et al., 2012). Thus, older adults likely maintain functionally similar effective stiffness at the muscle-tendon level by altering muscle contraction behavior. Indeed, prior studies have revealed age-related differences in soleus activation patterns (Schmitz et al., 2009) and fascicle behavior under matched walking speed conditions (Panizzolo et al., 2013). There is evidence that older adults utilize co-activation of the soleus and tibialis anterior to modulate ankle stiffness in mid-stance (Schmitz et al., 2009), which may in part be a compensation for increased tendon compliance.

There were age-related distinctions in how triceps surae work was modulated with speed. Young adults increased net soleus work by not only doing more positive work, but also doing less negative work at faster speeds. Older adults, however, did not show this trend and instead exhibited similar negative work across speeds. Ultrasound studies show soleus force-elongation behavior in stance tends to correspond with isometric behavior at the fascicle level (Cronin et al., 2013). Age-related increases in mid-stance soleus activity (Schmitz et al., 2009) may contribute to the relatively greater amounts of negative work at the faster walking speeds observed in this study. Speed-related distinctions in the work loop curves (Figure 2) emerged as the muscle-tendon reached peak stretch in stance and began shortening. Muscle fascicles are rapidly shortening in this period, with the rate of shortening increasing with walking speed (Lai et al., 2015). There are thus two possible contributors to the age-dependent deficit in positive work in this phase. First, older adult muscles may simply be less capable of generating force at higher contraction rates (Thelen, 2003). Second, an increase in tendon compliance could necessitate faster muscle shortening as the tendon stretches and then recoils during push-off. Further investigations that couple shear wave tensiometry with imaging of muscle-tendon interactions could provide insight into the relative influence of these factors.

Some notable assumptions and limitations should be considered. Subject-specific calibration was used to transform the wave speed measures to Achilles tendon loading. Our calibration approach for estimating tendon force from ankle torque relied on assumptions about muscle load sharing (Ebrahimi et al., 2020). While we did use EMG measures to ensure co-contraction was not present, we did not account for plantarflexors other than the triceps surae and this likely resulted in a slight overestimate of Achilles tendon force. We also measured subject-specific moment arms of the Achilles tendon, but our gastrocnemius excursion estimates relied on generic knee moment arm measures from the literature (Buford et al., 1997). We acknowledge that the standing sway calibration inherently produces a relatively lower force than arises during typical gait, and extrapolation is necessary to calculate tendon loading during gait. For example, max Achilles tendon forces during the sway calibration used here were an average of 46% lower than the peak Achilles tendon total forces walking at 1.25 m/s. We distributed net triceps surae load based on relative physiological cross-sectional area of the gastrocnemius and soleus (Hof et al., 2002). However, it is possible that the load distribution between triceps surae muscles varies between individuals and over time. Hence while our tensiometer approach allows us to characterize the net force and work done by the Achilles tendon, there remains uncertainty when decomposing the contributions of individual muscles. Our study analyzed muscle work patterns under controlled laboratory conditions which may not reflect real-world behavior. We are currently working on a portable shear wave tensiometer that could be coupled with wearable kinematic sensors to characterize muscle-tendon behavior during real-world gait. Several comparative biomechanists have effectively used similar coupled kinematic-kinetic approaches to understand animal muscle-tendon mechanics during real world locomotion (Roberts et al., 1997; Dickinson et al., 2000; Biewener et al., 2004; Daley et al., 2009).

In conclusion, this study demonstrates the novel use of tensiometry to characterize differences in triceps surae behavior during walking. Our results show that age-related deficit in triceps surae work production are evident even at slow walking speeds. These observations suggest that aging is associated with a fundamental decline in the reliance on the triceps surae to power walking and that this shift seems unrelated to the absolute mechanical demands of the task.

## Data Availability Statement

The datasets generated for this study are available on request to the corresponding author.

## Ethics Statement

The studies involving human participants were reviewed and approved by University of Wisconsin-Madison Health Sciences Institutional Review Board. The patients/participants provided their written informed consent to participate in this study.

## Author Contributions

AE, JM, and DT designed the study. AE collected and analyzed data, and prepared original draft and created figures. AE, JM, DS, and DT interpreted data. JM, DS, and DT reviewed, edited, and contributed to text. DT secured funding. All authors contributed to the article and approved the submitted version.

## Conflict of Interest

JM and DT are co-inventors on a patent for tensiometer technology (U.S. Patent No. 10631775). The remaining authors declare that the research was conducted in the absence of any commercial or financial relationships that could be construed as a potential conflict of interest.
